# Mechanically produced schistosomula as a higher-throughput tools for phenotypic pre-screening in drug sensitivity assays: current research and future trends

**DOI:** 10.1186/s40364-016-0075-2

**Published:** 2016-11-22

**Authors:** Emmanuel Mouafo Tekwu, William Kofi Anyan, Daniel Boamah, Kofi Owusu Baffour-Awuah, Stephanie Keyetat Tekwu, Veronique Penlap Beng, Alexander Kwadwo Nyarko, Kwabena Mante Bosompem

**Affiliations:** 1 Noguchi Memorial Institute for Medical Research (NMIMR), College of Health Sciences, University of Ghana, PO Box LG581 Legon, Accra, Ghana; 2 Laboratory for Tuberculosis Research and Pharmacology, Biotechnology Centre, Nkolbisson, University of Yaoundé 1, Yaoundé, Cameroon; 3 Centre for Plant Medicine Research (CPMR), Akwapim, Mampong, Ghana; 4 Yaounde Emergency Centre (CURY), Yaoundé, Cameroon; 5 School of Pharmacy, College of Health sciences, University of Ghana, Accra, Ghana

**Keywords:** Schistosomiasis, Newly Transformed Schistosomula, Mechanical transformation, In vitro drug sensitivity, Drug discovery

## Abstract

It is crucial to develop new antischistosomal drugs since there is no vaccine and the whole world is relying on only a single drug for the treatment of schistosomiasis. One of the obstacles to the development of drugs is the absence of the high throughput objective screening methods to assess drug compounds efficacy. Thus for identification of new drug compounds candidates, fast and accurate in vitro assays are unavoidable and more research efforts in the field of drug discovery can target schistosomula. This review presents a substantial overview of the present state of in vitro drug sensitivity assays developed so far for the determination of anti-schistosomula activity of drug compounds, natural products and derivatives using newly transformed schistosomula (NTS). It highlights some of the challenges involved in in vitro compound screening using NTS and the way forward.

## Background

Hundreds of millions of people are living at risk of schistosomiasis infection [1]. More than 207 million people are infected worldwide with 85% living in Africa. This makes schistosomiasis one of the most devastating tropical diseases in the world and remains a major source of morbidity and mortality for developing countries, especially in Sub-Saharan Africa [1]. Also known as “snail fever”, schistosomiasis is a water-borne trematodiasis carried by fresh water snails infected with one of the five varieties of the parasite *Schistosoma*. But three principal varieties are mainly the causative agents for human schistosomiasis: *Schistosoma mansoni*, *Schistosoma haematobium* and *Schistosoma japonicum*. The pathophysiology associated with schistosomiasis, is mainly due to immune response to the schistosome eggs that are trapped in tissues and organs. The liver, intestines and bladder usually trap eggs on their way out of the host. The spleen, as a lymphoid organ, becomes enlarged (splenomegaly) and together with the enlargement of the liver result in hepatosplenomegaly. Schistosomiasis has both an acute and a chronic phase. Acute schistosomiasis are generally short-term and mild and can develop a few weeks after the schistosome parasite first penetrates into the skin of the host. But if left untreated, schistosomiasis cause by any of the three species listed above may become a chronic inflammation which develops slowly into swelling, fibrosis and necrosis of the affected tissues such as intestinal organs, the liver and the bladder, as well as a wide range of other symptoms which gradually damage the host physiologically and even cognitively [2, 3].

Mass drug administration (MDA) in endemic areas using praziquantel (PZQ) remains a major cornerstone of schistosomiasis control programs [4]. Praziquantel was discovered in the year 1970’s and brought to the market in 1988 under the name Biltricide [5, 6]. It is so far the only drug available and recommended by World Health Organization (WHO) for the treatment of schistosomiasis. This single drug PZQ, is used for the treatment of millions of people annually and as stated in the recent publications, its coverage is projected to reach 235 million people by 2018, which raises concerns of increasing drug pressure [7, 8]. Furthermore, although there are several advantages of this drug, in particular its high efficacy on the adult worms of all the medically important *Schistosoma* species and its excellent tolerability, PZQ has some disadvantages, mostly its inefficiency against younger stage of schistosomes [9]. This mean that treatment does not rule out all the worms in those who are infected and necessitates the repeat of the treatment. Again, reliance on a single drug as the sole treatment, while positively reducing morbidity has led to big concerns over development of potential drug resistance [10]. These facts emphasize the imperative to search for the next generation of antischistosomal drugs. Therefore, a number of studies have recommended repeatedly the need for novel drugs, since the drug discovery and development pipeline is virtually dry [11]. Only a few candidates compounds have been studied in preclinical phases [12] and none of them have reached the clinical trials phase. For instance, the target product profile for a new antischistosomal drug [12] was not met by mefloquine and artemisinins [13, 14].

Formerly, procedures established at TDR-designated compound screening centers relied on adult worms incubated with the candidate drugs for 72 h [15]. Following the incubation period, the parasite viability was assessed microscopically [15]. This approach of in vitro drug screening based on the phenotype of the whole adult worm organism usually requires the intensive use of laboratory animals (hamster, rats, mice) since there is no existing in vitro life cycles for *Schistosoma*. This approach is also time consuming and low-throughput and relied on a small number of research groups experts that are able to handle the complex life cycle of *Schistosoma* and work with both high recovery of adult parasite from mammalian host and long screen timelines. The latter is the consequence of the long period that *Schistosoma* infection requires to become obvious since in the mouse model, it may take not less than 30 days for *S. mansoni* infections to become patent [16] and even more than 30 days for others specie such as *S. haematobium*.

Not long ago, the screening method using Newly Transformed Schistosomula (NTS) has been popularised as a higher-throughput [17–20]. Here, we have provided an overview for the alternative approach to phenotypic screening younger parasites that can be easily obtained from the intermediary host snails and in greater numbers than adult worms. This review presents the current state of in vitro drug sensitivity assays developed so far for the determination of anti-schistosomula activity of drug compounds, natural products and derivatives using NTS and highlights some of the challenges involved in in vitro compound screening using NTS.

## In which stage of the *Schistosoma* life cycle is schistosomula found?

**Fig. 1:**
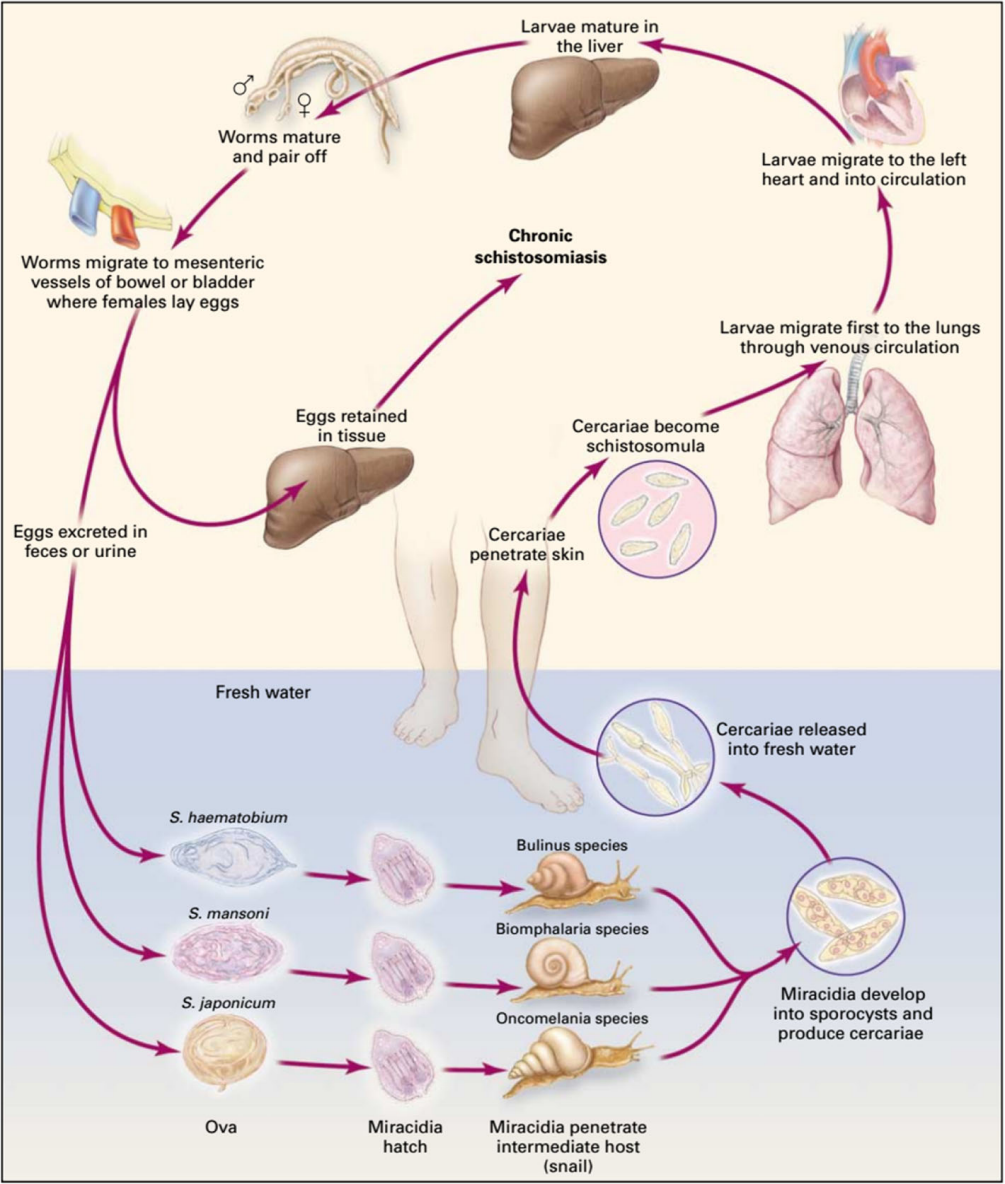
Life cycle of *Schistosoma* (Source: [22])

Schistosomes have a complex life cycle (Fig. 1). The transmission of schistosomiasis occurs when people harboring the parasite contaminate freshwater sources (lakes, ponds, rivers and dams) inhabited by snails with their urine (for urogenital Schistosomiasis) or faeces (for intestinal schistosomiasis) containing parasite eggs. Under optimal conditions the fertilized eggs hatch in water and miracidia are released. Once released from the eggs, miracidia swim in the fresh water and penetrate specific intermediate snail hosts. In the snail host, the miracidiae multiply by asexual division of 2 generations of sporocysts (primary and then secondary sporocysts) and produce cercariae from 4 to 6 weeks [21, 22]. Upon release from the snail, the infective cercariae can remain infective in freshwater for 1 to 3 days [22]. They swim in the water in search of the definitive host. Upon contact with the host, cercariae penetrate the skin of the host, and lose their bifurcated tail to become schistosomulae [22]. It takes several days for the schistomules to move from the skin into the venous circulation then into the lungs [22]. This path generally takes within 5 to 7 days after penetration. The schistosomulae takes at least 15 days to travel through the circulatory system to the hepatoportal circulation where they grow and mature into adult worms and pair up. In humans, adult worms are found to reside in either the perivesicular or mesenteric venules in various locations. This seems to be specific for each species. Specifically, *S. japonicum* occurs frequently in the superior mesenteric veins draining the small intestine while *S. mansoni* is frequently found in the superior mesenteric veins draining the large intestine. Nevertheless, *S. japonicum* and *S. mansoni* can occupy either location, and they are capable of moving between both sites, therefore it is impossible to state unequivocally that one species is specifically found in one location. Generally, *S. haematobium* is found in the venous plexus of the bladder, but can sometime be found in the rectal venules. The females start laying eggs from 4 to 6 weeks after infection in their final infection site which is either the bladder or the intestine. This usually continues for 3 to 5 years equivalent to the lifespan of the worm [22]. Eggs are laid in the small venules of the portal and perivesical systems. For *S. mansoni* and *S. japonicum,* eggs gradually move towards the lumen of the intestine and of the bladder and ureters for *S. haematobium*, and are excreted from the body through feces or urine, respectively [22]. The intermediate host snails are primary for transmission, and human contact with fresh water that harbors the infective larval form of the parasite known as cercariae is thus necessary for Schistosome infection [22].

## Importance of in vitro drug screening using Newly Transformed Schistosomula (NTS)

The in vitro culture techniques were developed for parasites after the successful establishment of the life cycle of *S. mansoni* in the laboratory [15]. The process relied on adult worms incubated with the candidate drugs for 72 h after which the viability of the parasite is microscopically assessed [15]. This approach of phenotypic screening using whole worm organism requires the intensive use of laboratory animals such as hamster, rats and mice since currently there is no existing in vitro procedure to generate adult worms. Moreover, this method is time consuming and low-throughput. It is no wonder that, in the recent years, researchers are using the Newly Transformed Schistosomula (NTS) as a tools for higher-throughput screening for drug sensitivity assay in the field of antischistosomals drug discovrey [12, 17–20, 23–27].

The era of working with schistosomula began with the recovery of mature schistosmulae from the lungs of infected laboratory animals. This was associated with the inconvenience of recovering only limited worm [19]. The transformation of cercariae into schistosomula using simple techniques as centrifugation, repeated aspiration through a syringe needle or chemical stimulation was an important discovery in the field of schistosome cultivation. Nowadays, researchers use cercariae as the starting material for in vitro studies of schistosomes as large numbers of schistosomula can be easily obtained in a manner which is advantageous in economical point of view [18, 19]. Furthermore and most importantly, the use of mechanically transformed schistosomulae limits and replaces the use of live animals in accordance with the 3Rs (reduce, replace, refine) principles of animal protection. Using an artificially produced schistosomula in in vitro drug sensitivity assays might serve as pre-screen tools.

## Changes associated with cercariae/schistosomulum transformation

*Schistosoma* infection occurs when people get in contact with free-swimming larval forms of the parasite (cercariae) released by freshwater snails. Cercariae, with the aid of its tail swim in the water until they penetrate the skin of the definitive host during contact with the contaminated water. From its intermediate snail host to the definitive host (human), the cercariae undergoes a series of adaptive changes. All these changes are known as transformation. During the transformation process, the tail of cercariae is lost and the secretory glands release two substances: a mucus which promotes attachment to the skin and enzymes which degrade the skin [28]. Moreover, on the surface of schistosomula, appears the transient microvilli and a double unit membrane is formed on the tegument [29]. Concurrently, some of the glycocalyx is lost [29]. McLaren and Hockley reported that in vivo, microvilli are developed on the surface of schistosomula obtained throughout penetration of the host skin. This also occurs on schistosomula which have penetrated a mouse skin prepared in vitro and on artificial schistosomula prepared by mechanical separation of the tail from the head of the cercariae [30]. From their investigation, they reported that microvilli occur at approximately the same time, have about the same life-span and show identical morphological characteristics in each of the three types of schistosomula [30].

Schistosomules rapidly undergo marked physiological and ultrastructural changes in the body of the definitive host in order to adapt to the host’s internal environment [31]. Some of these changes are loss of the glycocalyx, conversion of trilaminate to heptalaminate tegumental membrane [29], sensitivity of schistosomula to water (loss of water tolerance) [28], and evacuation of secretory glands. All these changes result in the schistosomulum stage. Transformation is complete within about 2–3 h [30, 32]. Brink et al. published in 1977 a study conducted to compare schistosomula produced artificially and schistosomula recovered after cercariae had penetrated isolated skin [33]. They concluded that the schistosomula prepared by mechanical separation of the tail from the head of the cercariae fulfill the main criteria of transformation from cercarial to schistosomulum. This is justified by the fact that, the surface membrane of all type of schistosomula (those mechanically obtained and those recovered after cercarial penetration of the isolated skin) had changed from trilaminate to heptalaminate structures and had loss their cercarial glycocalyx within 2 h of transformation [33]. They also reported that in vitro, the development of mechanical transformed schistosomules is similar to schistosomula obtained after cercarial had penetrated isolated skin although only 25–50% of mechanically transformed schistosomules reached the ‘gut-closed’ stage by day 12, while 50–70% of skin transformed schistosomules reached this stage [33]. Thus, 2 mains criteria were generated by the authors to help decide whether transformation from cercariae to schistosomula has been effective. The 2 criteria were, the NTS must have developed heptalaminate surface membranes and they must be capable to growth and develop in vitro, at least to the ‘gut-closed’ stage [33].

## Requirement for in vitro drug screening using NTS

### Drugs and chemicals

Praziquantel, mefloquine, auranofin, artesunate, metrifonate, oxamniquine, artemisinins, arthemeter are drugs used as antischistosomal compounds. These drugs are usually dissolved in dimethylsulphoxide (DMSO) to obtain drug stock solutions of 10 mg/ml or 10 mM and then diluted into culture media to serve as positive control [12, 20, 23, 25, 26, 34]. The highest concentration of DMSO used as drug solvent should not exceed 1% [15, 18, 24, 34].

Antifungal drug (Amphotericin B) and antibiotic drugs (penicillin 100 to 300 U/ml and streptomycin 100 to 300 μg/ml) are used to supplement the medium in order to avoid fungal and bacteria contamination during schistosomula culture [12, 17, 23, 25–27, 34].

### Media

Different type of media such as Basch Medium 169 [18–20, 26, 27, 35, 36], Earles’Minimum Essential Medium [32, 33], Dulbecco’s Modified Eagle’s Medium (DMEM) [17, 19, 26, 31, 35, 37], Minimum Essential Medium (MEM) [19, 27, 35], Roswell Park Memorial Institute (RPMI 1640) [18, 31, 32, 38] and Medium 199 [12, 19, 23–26, 34, 35] have been used in different studies. Other investigations were developed to compare and obtain optimal culture conditions for the NTS. For the optimal culture condition for schistosomula, several studies have shown that the supplemented medium is appropriate. Different concentrations of heat inactivated fetal calf serum (iFCS) 1% [24], 5% [12, 20, 23, 25–27, 34, 38], 10% [17, 37], or heat inactivated fetal bovine serum (FBS) 5% [38], 10% [36] have been used.

The supplemented medium 199 turned out to be the most suitable medium for the incubation of schistosomula and thus has been widely used for in vitro screening of others compounds with known activities against *Schistosoma* newly transformed schistosomula [12, 23–25, 34]. Thus 5 to 10% heat inactivated calf serum is the commonly used supplement by several researchers.

### Snails and cercariae

**Fig. 2:**
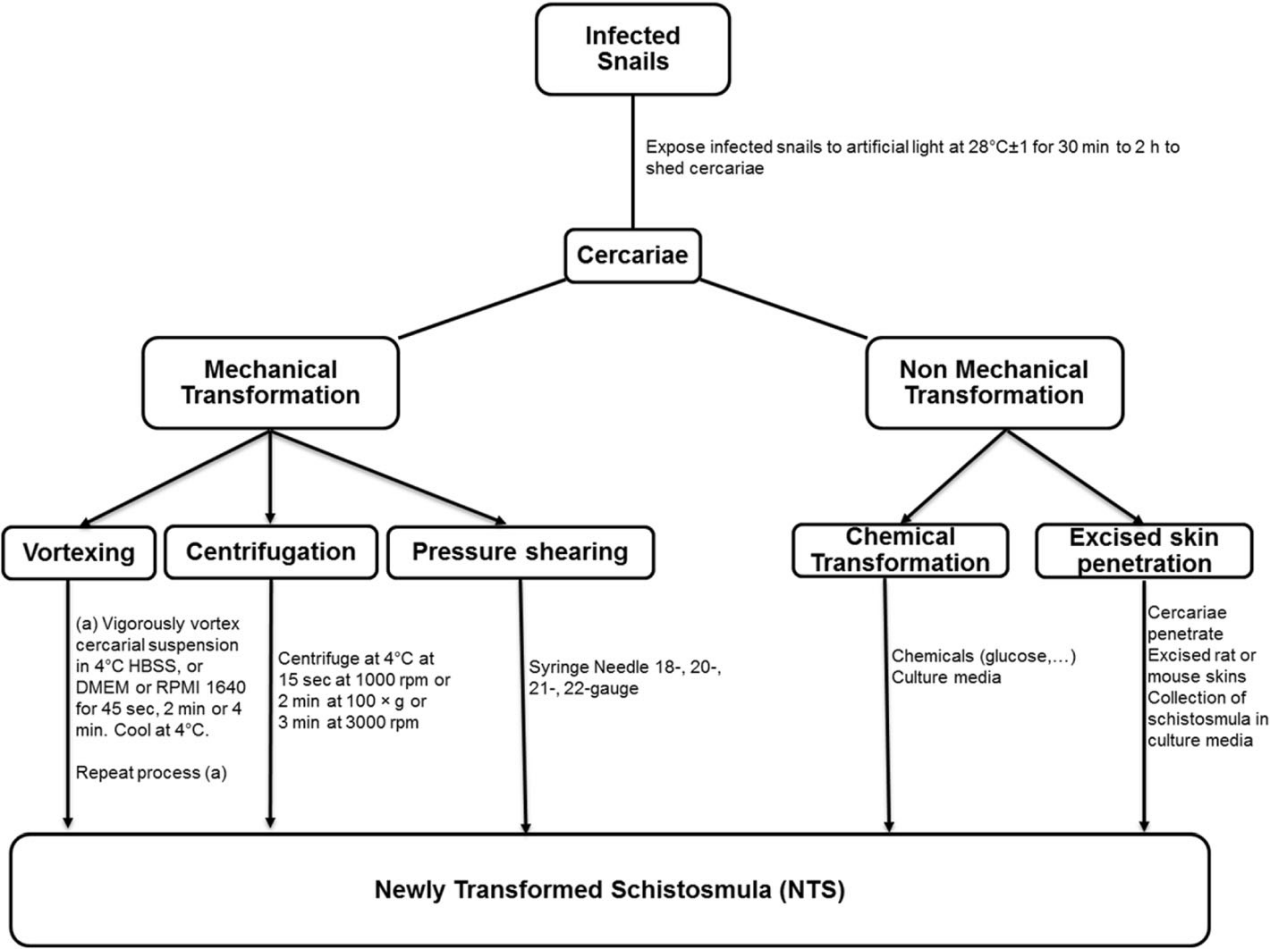
Flowchart of Newly Transformed Schistosomula (NTS) Procedure

The snails might be of the genus *Bulinus* which serves as the intermediate hosts of *S. haematobium* as well as of *S. intercalatum* or the genus *Biomphalaria* or *Oncomelania* for *S. mansoni* and *S. japonicum* respectively. Snails are individually infected with an average of 10 miracidia per snail [26]. In our laboratory, an average of 5 can be used to successfully infect snails. Usually, snails are kept in aquarium with dechlorinated water in a humid room simulating a 12 h day and night cycle. Intermediate host snails start shedding cercariae from 4 to 6 weeks post-infection with miracidia. According to the circadian rhythm, each snail species are then collected and placed individually into 24 or 48 well plates or in a test tube (1 ml of distilled or dechlorinated tap water/well or per tube). Each test tube or well plate can be exposed to artificial light for 30 min [39], 1 h [17], or 2 h [40–43]. The cercarial suspension is then collected, cleaned and concentrated by allowing to stand on ice for 30 to 60 min. During this time, cercariae form into a mass, settle and adhere to the bottom of the tube. The supernatant is poured off and replaced with ice-cold distilled water. This suspension is used for the preparation of schistosomula. There are several techniques for transforming cercariae into schistosomula and maintaining them [18, 33, 44–46]. Some of these techniques are performed in vivo and others in vitro. In vivo transformation occurs after host skin penetration [33] and in vitro transformation can be obtained when parasites penetrate excised skin [33, 47] or when cercariae tails are removed mechanically [12, 18–20, 23–25, 27, 33, 34, 45] or chemically [26] and cercariae bodies are incubated in physiological media (Fig. 2).

### Newly transformed schistosomula (NTS)

#### In vitro transformation of Schistosome cercariae to schistosomula and maintenance

As the schistosomulum stage is becoming attractive for search of new antischistosomal drugs, this requires the development of efficient, reproducible and rapid means to generate large and suitable quantities of the biological material. The artificial transformation of cercariae into schistosomula can be induced by various effectors for instance the cell growth media at 37 °C [48, 49] or low osmolarity phosphate buffer saline solution [50] that seem to be able to start off the transformation of cercariae into schistosomula. In vitro, schistosomula can be obtained through excised skin preparation or by mechanical separation of the tail from the body of the cercariae [30]

#### Non-mechanical transformation

##### Excised skin preparation of schistosomula

Schistosomula can be prepared from cercariae of schistosome by allowing them to penetrate excised rat [51] or mouse [52] skins into Hanks' Balanced Salt Solution (HBSS). The possibility of contamination by host material and the low yield of produced schistosomula as reported by Brink et al. [33] make the excised skin penetration technique inappropriate.

##### Chemical transformation using glucose

The cercarial transformation can be performed using glucose. This is known as chemical transformation which is carried out based on protocol previously described [45]. Briefly, the cercariae suspension is cooled on ice for 30 min to reduce parasite motility. Afterwards, the cercariae suspension is centrifuged for 2 min at 2000 rpm, then resuspended in 5% glucose and incubated for 10 min at 30 °C [26]. The tails are removed from the bodies using the ice purification method as described below.

#### Mechanical preparation of schistosomula

The mechanical transformation protocol is the most popular method for obtaining artificially transformed schistosomula. This method is applied to freshly shed cercariae and includes centrifugation, passages through an emulsifying needle, or shaking. The separation of cercariae body from tails is usually done by centrifugation in a density gradient followed by incubation of the cercariae heads in culture media at 37 °C. In order to detach the tails from the body, these methods usually incorporate an initial step consisting of agitating the organisms sufficiently. Schistosomules prepared in vitro and incubated at 37 °C gradually undergo morphological and physiological changes [31]. Tucker in 2001 [31] reported that mechanically transformed schistosmula by 24 h in culture, resemble in most respects cercariae that have penetrated and resided in the skin for about 1 h. Thus, parasites obtained following this protocol is morphologically or biochemically closed enough to those recovered from natural infections [33, 48]. This makes the mechanical transformation the best alternative method for large production of schistosomula for high-throughput studies such as gene expression, identification of drug targets and identification of effective drugs against schistosmes [37].

In this paper, we will describe the two of the three mechanical methods that have been employed for the acquisition of schistosomules. These include centrifugation, vortexing, or pressure shearing, such as using the 18-, 20-, 21-, 22-gauge–needle method [31, 37, 43–45]. The double-ended–needle method that presumably was developed recently, from the normal needle process seems to be the most widely used for cercariae transformation in vitro [31, 35, 37, 38, 43, 44].

##### In vitro transformation of cercariae to schistosomula by vortexing

The transformation of cercariae to schistosomula by vortexing is performed based on a protocol of Ramalho-Pinto [45]. Many laboratories used a slightly adapted Ramalho-Pinto method [19, 26]. Briefly, cercarial suspension is cooled on ice for 10 min [33], 30–40 min [26, 31] in order to reduce parasite motility. Then cercariae are further concentrated by centrifuging for 15 s at 1000 rpm [33], 2 min at 100 × g [31], 3 min at 3000 rpm [26], 4 °C. The supernatant is discarded and the cercarial pellet is resuspended in cold Hanks` Balanced Salt Solution (HBSS) containing or not penicillin-streptomycin and amphotericin B [18, 26], or in 4 °C DMEM or RPMI 1640 [31]. The suspension is vigorously vortexed for 2 min [27], 4 min [26], or 45 s twice separated by ice cooling for 3 min [31] in order to start off tail loss. This step is repeated after an incubation of the mixed tail-schistosomula suspension for 20 min at 37 °C [26]. Tucker [31] recommended addition of antibiotics if incubation following the transformation will be longer than 8 to 12 h.

##### In vitro transformation of cercariae to schistosomula by needle and syringe

Using a needle and syringe to separate the tails from the head of cercariae is an equally valid method for the initial phase of preparing schistosomula. In this case, cercarial suspension is placed into a plastic centrifuge tube and a syringe with 18-, 20-, 21-, or 22-gauge–needle is filled. The cercarial suspension is repeatedly passed through the needle (10–20 times back and forth) [31, 38, 43–45].

For the double-ended–needle method, an 18-, 20-, 21-, or 22- gauge double-hub, emulsifying needle with a stabilizing bar is fitted to a sterile 10 ml syringe [43]. Cercariae are drawn up using a second 10 ml syringe which is fitted to the open end of the emulsifying needle. The cercariae tails are sheared by approximately 10–20 passes back and forth through the needle [31, 37, 38]. This procedure involves manipulation of thousands of cercariae which is an obvious biohazard. Therefore mandate the use of protective clothing and gloves, including protection for the face and the eyes. Afterwards, the schistosomule bodies are isolated from the sheared tails by Percoll gradient centrifugation [53] or another purification method as described below. Table 1 compare the different schistosomula preparation procedure with advantages and disadvantages.

**Table 1:** Advantages and disadvantages of different ways for preparing schistosomula

	Advantages	Disadvantages
Mechanical methods (Centrifugation, Syringe needle method, Vortexing)	Relatively easy and Inexpensive	Increased parasite damage
	Manipulation of thousands of cercariae	Increased risk of infection to researchers (Potential biohazard to the researcher)
	Replaces the use of live animals	
	Help to obtain large number of schistosomula	
	Morphological characteristics identical to schistosmula obtained naturally	Only 25–50% of transformed schistosomula reach the ‘gut-closed’ stage by day 12
Non-mechanical methods (Chemical transformation & Excised skin penetration)	Ideal alternative to obtaining high numbers of viable schistosomula	Significantly less cercariae heads separated from the tails by chemical method
	Simpler Less damaging to the parasites	Low schistosomula yield and the possibility of contamination by host material
	50–70% of skin transformed schistosomula reach the ‘gut-closed’ stage by day 12	Require use of live animals (rat, mice, hamster, …) and skilled technician
	Schistosmula are obtained naturally	Less appropriate technique for high throughput

#### Purification of newly transformed schistosomula (NTS)

The schistosomule bodies can be isolated from the sheared tails by three purification methods which are Percoll, ice and swirling method.

The Percoll method is based on the method of Lazdins et al. [53]. The separation of the bodies from the tails is done on a 70% Percoll gradient (polyvinylpyrolidone-coated colloidal silica particles) by centrifuging for 15 min at 500 × g, 4 °C [31]. After centrifugation, the sample is collected from the bottom of the tube and the collected fraction is then diluted to the culture medium (RPMI 1640, DMEM, Medium 199, 169, MEM, etc…) and centrifuged [31, 37, 38]. The schistomula pellet is resuspended in fresh, warm supplemented medium.

Another purification method of schistosomula is a swirling technique which is a simple and easy. This method was described previously [31, 38]. In this case, schistosomula suspension is poured into a Petri dish with a sufficient warm medium such as 199 [26], 37 °C RPMI 1640 complete media [38] or incomplete medium 169 [18]. By swirling the dish gently, all the bodies are settled in the centre and the lighter tails can be aspirated and the bodies are left accumulated in the centre. The bodies (schistosomulae) will be further transferred into 15 or 50 ml centrifuge tubes. This step (swirling and collecting) is repeated until schistosomula are no longer present in the center of the dish. This objective can be reached approximately after 4 to 5 times [26] or 10 times [38].

For the ice method, 7 ml of cold HBSS is added to the schistosomula suspension and cooled on ice for 7 min. The supernatant is decanted, and the pellet resuspended again in 7 ml of cold HBSS. This step is repeated three times. The pellet that contained the recovered schistosomula is then resuspended in pre-warmed (37 °C) supplemented Medium 199 [26] or Basch medium [27].

The obtained purified schistosomula are kept in the schistosmula culture medium and incubate at an atmosphere of 37 °C of 5% CO_2_ for further experiments.

After the purification, the transformation rate and the purification factor can be estimated. The rate of transformation is estimated by counting the total number of cercariae in the HBSS suspension before transformation, and placing them in relation to the total number of schistosomulae obtained after purification [26]. Marxer et al. [26] reported a transformation mean rate of 69% for five identically performed mechanical transformations by vortexing and 34% for the chemical transformation.

To estimate the purification factor, the total number of bodies and tails are counted in a sample of 50 μl after the experiment. The ratio is expressed as purification factor. Marxer et al. [26] after performing the three purification methods, they calculated the mean purification factor for each of the methods. The best purification method according to their analysis is Percoll with purification factor of 24.4 ± 11.4 [26]. This method was followed by the swirling method with a purification factor of 11.7 ± 3.2 [26]. The ice method presenting a very weak mean purification factor of 3 ± 1.7 [26].

## Schistosomula culture and culture media

Techniques required to cultivate parasites in vitro is a major area of concern among present day researchers. Aside the dynamic study and understanding the physiology, behavior and metabolism of parasites, the nature of the antigenic molecules found in their excretory and secretory products need to be vigorously pursued and analyzed upon a successful establishment of the in vitro culture of the parasite. However, this is a difficult task since parasites have complex life-cycles comprising different stages and host species requirements. A good mention is in the case of parasitic helminths. Techniques involved in parasites culture requires knowledge of all types of microbiological cultures as different parasites demands particular culturing conditions such as nutrients, temperature and even incubation conditions.

**Fig. 3:**
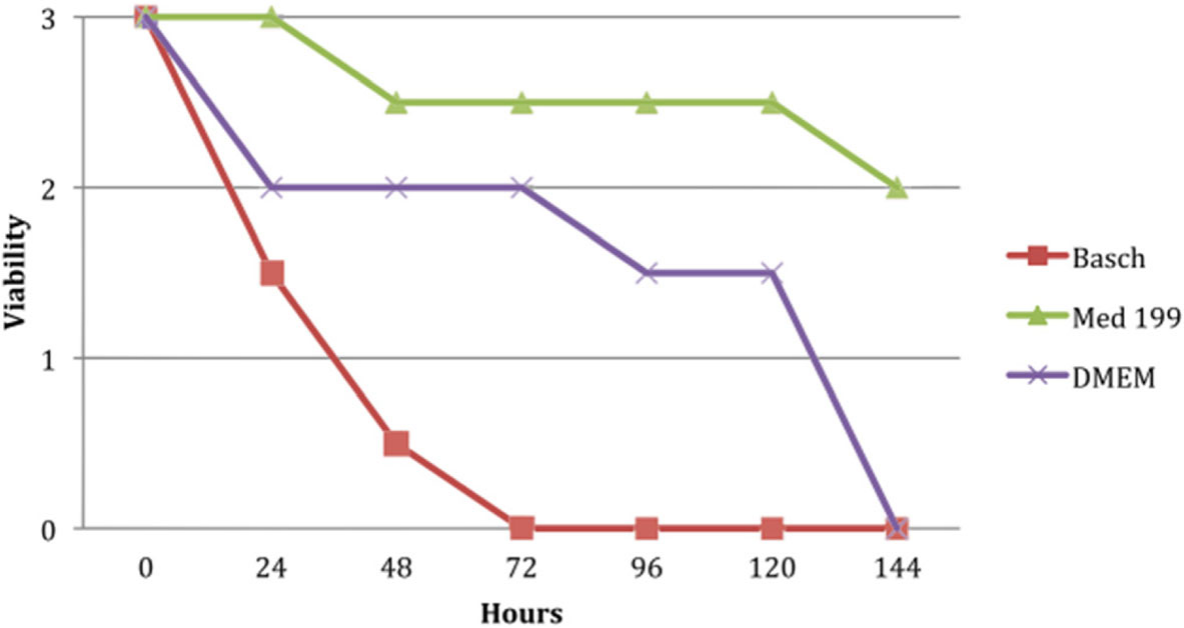
Survival time of *S. haematobium* NTS in different culture media (Source: [26])

Cultivation of parasites has immense usefulness in the production of vaccines, testing efficacy of vaccine, and production of antigens for obtaining serological reagents, detection of drug-resistance, screening of potential therapeutic agents and conducting epidemiological studies. Parasite cultivation is always a challenge. In the case of *schistosoma*, transformed schistosomula can be grown and maintained in vitro in a complex medium [35]. The growing rate of schistosomules cultured in vitro is not the same as those in a permissive host, nor will they become patent adults. Using good and appropriate methods, about 50% of the cultured parasites will mature with fully formed guts, and 10% will develop into sexually distinct male and female worms [31]. Starting with good number of parasites, a good amount of worms can be easily maintained and provide a vast quantity of parasite material for assay. There is also a possibility to increase the percentage of schistosomules forming guts and growing properly (50% versus 20%). This can be achieved by supplementing the growth media with conditioned media during the first week of culture [31]. Using the method described by Tucker et al*.* [31] with supplemented DMEM or RPMI1640 media, *Schistosoma spp.* schistosomules can be grown in culture media for at least two months. Abdulla et al*.* [18] noted that the Basch Medium 169 is chosen over RPMI for schistosomula culture since worms survive more in the Bash Medium with 10% mortality for up to 4 weeks whereas in RPMI medium, an average of 40 to 60% of the parasites die within 3 days with continued mortality up to two weeks . Keiser in 2010 reported that schistosmula can survive for at least 96 h in different culture media such as MEM, DMEM, Basch, or TC 199 [19]. Marxer et al*.* [26] showed that supplemented Medium 199 is most suitable for the incubation of schistosomula obtained by transformation in supplemented HBSS. After undertaken comparison study on three culture media (DMEM, Medium 199, Basch medium), they reported that in the supplemented Medium 199, the parasites were still alive after 120 h with an average viability value of 2.5 while all schistosomula died by 72 h in Basch medium and by 144 h in DMEM on the other hand as shown in Fig. 3 [26]. Schistosomules are cultured at 37 °C in a 5% CO_2_ incubator [12, 17, 20, 23, 25–27, 31, 34, 37, 43, 45, 54]. It was also shown that over the course of one week’s incubation, parasites in Basch Medium 169 appear robust and uniform in shape and appearance. The effects of culture media on the growth of schistosomula are summarized in Table 2.

**Table 2:** Effects of different culture media on the growth of schistosomula

Culture Media	Shape and appearance of NTS	Motility of NTS	Duration/Viability of NTS
Basch Medium 169	Parasites (NTS) appear robust and uniform	High dynamic motility	Keeps parasites alive up to one week’s incubation. Worms survive more with 10% mortality for up to 4 weeks
DMEM	Parasites (NTS) appear distorted including rounding and darkening	Slowed and less dynamic motility	Keeps parasites alive up to 144 h When the culture media is appropriately supplemented schistosomula can be grown for at least two months
Medium 199	Generate different degrees of distorted parasites (NTS) including rounding and darkening	Slowed and less dynamic motility	Supplemented Medium 199 keeps the parasites alive up to 120 h with an average viability value of about 2.5
RPMI 1640	Parasites (NTS) rounding	Slowed and less dynamic motility	Parasites degeneration and average death of 40 to 60% within 3 days with continued mortality up to two weeks When the culture media is appropriately supplemented schistosomula can be grown for at least two months

*NTS* newly transformed schistosomula, *DMEM* Dulbecco’s modified eagle’s medium, *RPMI* Roswell Park Memorial Institute

Although the more suitable medium vary from one study to another, we can observe that studies initiated to screen compound FDA library [25], Medicines for Malaria Venture (MMV) box compounds [12], Mefloquine-related Arylmethanols [34] made used of supplemented Medium 199. Thus the supplemented medium 199 seems to be the most suitable medium for the cultivation of schistosomula and might justify why it has been widely used for in vitro screening of other compounds with known activities against *Schistosoma* newly transformed schistosomula [12, 23–25, 34].

The biggest obstacle to a successful schistosomules culture is contamination by fungal and bacterial, which is mainly due to the fact that the parasites originate from non-sterile snails. Therefore, the use of antibiotics (Penicillin and Streptomycin) is useful to prevent bacteria growth and amphotericin B, a fungizone that prevent fungal growth. Different concentration as indicated above are used.

## In vitro drug sensitivity assays with newly transformed schistosomula

### Schistosomula screening

*Schistosoma* NTS in vitro Assay is obtained using one of the transformation methods already described in this review. The NTS suspension adjusted to a concentration of 100 NTS per 50 μL [12, 25, 26, 34] is then incubated in culture medium at 37 °C, 5% CO_2_ in ambient air for a minimum of 12 to 24 h to allow maturation or in order to achieve complete transformation into schistosomula before being further processed [12, 17, 20, 23–25, 34]. Other authors incubated NTS suspension just for 1 to 3 h before being used in subsequent experiments [18, 27]. The transformed schistosomula in schistosomula culture medium are then incubated with the test drugs in a 96-well flat bottom plate in duplicate or in triplicate and at least 2 to 3 time at a number of 100 NTS/well [12, 20, 25, 26, 34]. Since drug stock solution is prepared in DMSO, the highest concentration of DMSO diluted in Schistosomula culture medium is used to serve as control. Thereafter, drug effects are assessed microscopically under an inverted microscope.

### Determination of schistosomula viability in response to test compound

Based on microscope readouts, phenotypic changes of NTS is recorded at 3 different time points (24 h, 48 h, 72 h post-drug exposure). The changes are recorded with regard to death of worms, changes in motility, viability and morphological alterations [12, 25, 34]. This makes use of a viability scale from 0 to 3 (3 = motile, no changes to morphology; 2 = reduced motility and/or some damage to tegument noted; 1 = severe reduction to motility and/or damage to tegument observed; 0 = dead) [12, 13, 25–27, 34]. The 50% (IC50) and/or 99% (IC99) inhibitory concentration are determine for active compounds [12, 23–26, 34].

### Inconvenience of microscopic read-out and development of automated technologies

Current methods utilized to assess schistosomula viability encompass microscopic techniques. In this case, the experimenter manipulates the parasite in vitro and assesses the effect of such manipulation by bright field examination of morphology. This procedure has been used in several drug screening protocols [18, 25–27] and general manipulations of parasite development [55]. Schistosome viability has been assessed in various research work using different criteria. Some of the criteria are intracellular granularity, schistosomula movement, schistosomula shape alterations. Nevertheless, evaluating parasite viability through microscopic read-out are itself potential deterrents to the development of high throughput since these methods are slow and subjective and therefore represent a bottleneck for high-throughput screening. More often, high throughput methods are dependent upon assay miniaturisation, objectivity and very demanding in term of quantification. Several complementary techniques initially developed for single cell eukaryotes viability measurement have been adapted to multicellular schistosomes parasites. Among the existing viability assays that have been developed for use with single cell eukaryotes, it is amazing to find that only a limited range of techniques have successfully been translated to studies with schistosomes. This may be explained by the multicellular nature of schistosomes which size more than 1 cm with complex tissue and also its external tegument bound by a heptalaminate membrane [56]. The heptalaminate is thought to be selectively permeable to macromolecules, simple compounds and water [57]. Nonetheless, there is a challenges due to parasite biology, there is also evidence that some techniques developed for single cell viability can successfully be adapted to schistosomes.

The size and complexity of schistosomes can partly be considered as useful attributes for determining viability. One of the valuable trait in assessing schistosome (both schistosomula and adult worm schistosome) viability in vitro is their regular movement, although lack of movement is thought to be not infallible indicator of death. Motility together with other microscopic characteristics such as morphology changes, granularity and tegument damaging [12, 25, 34] are currently the most common indicators for assessing schistosome viability and represent the “gold standard” for assessing drug screening protocols within the schistosomiasis research community [12, 18, 20, 25, 34].

Despite the wide application of bright-field, light microscopic assessment of schistosome viability, this technique presents several problems. Firstly, the personnel should be well trained to acquire sufficient knowledge on diverse schistosome phenotypes. Secondly, the bright-field, light microscopic detection of schistosome viability will always be subjective due to lack of immunological and molecular evidence that death has actually occurred when a schistosome is immobile. Even when the personnel has acquire the proficiency at identifying schistosome phenotypes, this technique is still slow and tedious. For instance a recent study performed the screening of only 640 potential anti-schistosomula compounds per month [18]. Finally, replication of results obtained by bright-field microscopic means is not always possible, because of the absence of uniformity between laboratories. With recent advancements in automated technologies, a number of alternatives read-out assay have been attempted with varying degrees of success in order to avoid the subjective nature of quantifying viability of schistosome from microscopic observation of phenotype alone [58]. For example, methylene blue has shown to stain differentially dead schistosomula and therefore was considered as a reliable dye for dead schistosomula [55] and has been used to assess the viability of mechanically transformed schistosomula [59]. Regardless, it was thought that vital dyes can be successfully translated from single cell viability markers to multicellular schistosomes.

Some fluorescent compounds such as DNA intercalating dyes, ethidium bromide (EB) and Propidium Iodide (PI) [60, 61], carboxyfluorescein [61] as well as resazurin have also been used to quantify schistosome viability in low-throughput, microscopic read-out methods. In more detail, resazurin, the active ingredient of Alamar Blue (AB), a non-toxic and cell permeable compound, is blue in color and virtually non-fluorescent. But when resazurin enters the cells, it is converted to resorufin which is red in color and highly fluorescent. Resazurin is continuously reduced to resorufin by viable cells. This staining technique was used to discriminate between live and dead schistosomula after 48 and 72 h of incubation with standard selected drug compounds of known antischistosomal activity [26]. Unfortunately this technique cannot be used for earlier time-points and to measure dose response drug effects [23].

Ethidium bromide has been used to differentiate dead schistosomula from live ones during microscopic examination [61], while PI has successfully been used for the same reason (as a differential stain of dead schistosomula) for both microscopic examination and flow cytometry [60]. Carboxyfluorescein in contrast to ethidium bromide and propidium iodide, has been tested as live staining for schistosomula. However, it was difficult to clearly differentiate live schistosomula to dead ones since the latter developed some fluorescence [61].

By promoting the use of single dye staining, a dual fluorescent viability assay has been developed for schistosomula [17]. In this case, authors combined the use of PI with fluorescein diacetate (FDA) to easily assess the percentage of viable schistosomula present in a sample. By using a microtiter plate reader, this fluorescent bioassay was developed for 96 or 384 well microtiter black-sided, flat-bottom plate optically clear. Ninety-six (96) well plate were designed for medium throughput and 384 for high throughput applications [17]. The use of the fluorescent bioassay has the added advantages that it could increase by 10-fold the number of compounds screened per month over existing microscope methodologies and also, it does not require extensive training of personnel in parasite morphology [58]. Therefore fluorescent bioassay has been shown to be entirely objective [58] and has been validated with schistosomula. Currently, there are indications that fluorescent bioassay can be adapted for use with adult worm schistosomes as well as other life stages. Although the combined use of PI and FDA, can objectively and rapidly quantify schistosome viability in a high-throughput format, the ability of PI and FDA to provide significant phenotypic data is slightly limited [58]. Therefore, there is still a need for the complementary technologies to the fluorescent bioassay and methodologies that allow the automated assessment of phenotype that could be seen as a great discovery in the field of novel antischistosomal drugs discovery.

Smout et al. [62] developed a motility assay that was thought to be one of the technological advance to offer a solution to this helminth phenotype quantification challenge, until high content screening as an affordable reality. This assay, which uses the xCELLigence system was a new application or device to monitoring cells in a real-time manner. This was known to simply and objectively assess anthelmintic effects by measuring parasite motility in real time in a fully automated high-throughput fashion. In principle, this technique is based on the detection of changing electrical currents running through mini gold electrodes incorporated into the bottom of tissue culture plates. When schistosomes in the immature and mature stages are quite dense, they usually sediment during in vitro culturing and this make contact with the gold electrodes. It is known that, changes to the culturing conditions that may impact a worm’s physiology will probably modifiy its behavior or phenotype. This result to a measurable fluctuation in current across the electrodes [58]. Since many anti-schistosomal drug compounds act by affecting the motility of the target parasite, the importance of these measurable current fluctuations can be considered as an indicator of potential therapeutic activity. This biophysical characteristic to assess anthelmintic activity of compounds in real time in a high-throughput fashion was demonstrated by Smout et al. [62]. This technology was applied to adult schistosomes to illustrate that by increasing the doses of PZQ, the signal decreased from adult worm schistosomes, and this allowed to generate a dose dependent curve. Can this technology be applied to larval schistosome life stages? Although the answer to this question is currently unknown, it appears feasible. It is believed that this assay may provide an advanced methodology to microscopy that will help remove subjectivity in helminth phenotype characterization as well as making available a technology to compare results directly from different laboratories [58]. But the original cost of the xCELLigence equipment may restrict its widespread use.

Howe et al. [24] investigated a fluorometric L-lactate assay for viability in *schistosoma* drug screening assays. Lactate is a by-product of glycolysis. It is secreted via aquaglyceroporins from NTS and adult worm schistosomes [23]. Authors fully investigated parameters of lactate measurement and performed drug sensitivity assays by applying schistosomulae and adult worms to establish a proof of concept. They showed that lactate levels reflect clearly the viability of schistosomula and this was also correlated with schistosomulae numbers. They tested compounds with described potencies, and compared activities of fluorometric L-lactate assay with microscopy. Howe et al. [24] concluded that lactate can be used as simple surrogate marker since its measurement can be a promising new approach to assess the viability of schistosomulae in drug sensitivity assays. However, this technique requires two things. Firstly, the supernatant must be removing from the drug assay without aspirating the Schistosomula and secondly, the drug assay should be diluted to an acceptable fluorescence range as needed. These two aspects make the fluorometric L-lactate assay less than high-throughput [23].

More recently, the commercial luminescence-based cell viability kit known as CellTiterGlo® was validated by Lalli et al*.* [63] for the in vitro assay using *S. mansoni* NTS and adult worms schistosomes. In this procedure unfortunately, a precise multi-drop dispenser is required to ensure an exact number of NTS present in each well. Although the investigation of marker-dye based assays has been a popular activity, we can simply note that, the aim of a simple, inexpensive and accurate dye that does not require much additional equipment or analysis has not so far been entirely met [23]. Table 3 summarizes different in vitro drug sensitivity assays developed so far for the determination of anti-schistosomula activity of drug compounds. The summary of publications cited in this review are contained in Table 4.

**Table 3:** In vitro drug sensitivity assays developed for the determination of anti-schistosomula activity of drug compounds

Methods	Principles	Advantages	Disadvantages
Microscope readouts without staining	Parasites are manipulated in vitro and the effect is assessed by bright field examination of morphology. Schistosomula viability is assessed using different criteria: - intracellular granularity - schistosomula movement - schistosomula shape alterations This makes use of a viability scale from 0 to 3	- Used to discriminate between live and dead schistosomula after incubation	- The personnel should be well trained to distinguish diverse schistosomula phenotypes - The bright-field, light microscopic detection of schistosomula viability is subjective due to lack of immunological and molecular evidence that death has actually occurred when a schistosomula is immobile - The technique is slow (time consuming) and tedious - Replication of results is not always possible, because of the absence of uniformity between laboratories
Microscope readouts with Staining using a single fluorescent dye	Parasites are manipulated in vitro and the effect is assessed by bright field examination of viable or dead cells	- Used to discriminate between live and dead schistosomula after incubation - Does not require extensive training of personnel in parasite morphology - The fluorescent bioassay is objective - Fluorescent bioassay can be adapted for use with adult worm schistosomes as well as other life stages	- Cannot be used for earlier time-points - Cannot be used to measure dose response drug effects - It is sometime difficult to clearly differentiate live schistosomula from dead ones
Microscope readouts with Staining using dual fluorescent viability assay	Combination of the use of DNA intercalating dyes (ethidium bromide (EB), Propidium Iodide (PI)) with Carboxyfluorescein (fluorescein diacetate), Resazurin to easily assess the percentage of viable schistosomula present in a sample	- This bioassay was developed for 96 or 384 well microtiter plate optically clear - Designed for medium throughput (96 well) and high throughput (384 well) applications - Increase by 10-fold the number of compounds screened per month over existing microscopy methodologies - Does not require extensive training of personnel in parasite morphology - The fluorescent bioassay objectively and rapidly quantify schistosomula viability in a high-throughput format - Fluorescent bioassay can be adapted for use with adult worm schistosomes as well as other life stages	- The ability of the dual fluorescent dye to provide significant phenotypic data is slightly limited
Motility assay	The assay uses the xCELLigence system for monitoring cells in a real-time manner. This technique is based on the detection of changing electrical currents running through mini gold electrodes incorporated into the bottom of tissue culture plates	- Simply and objectively assess anthelmintic effects by measuring parasite motility in real time in a fully automated high-throughput fashion - Help remove subjectivity in helminth phenotype characterization - Results can be compared directly from different laboratories	- The xCELLigence equipment used in this technology is costly and may restrict its applicability
Fluorometric L-lactate assay	Consist of the measurement of lactate levels that reflect clearly the viability of schistosomula and this also correlate with schistosomula numbers	- Can be used as simple surrogate marker - Promising new approach to assess the viability of schistosomula in drug sensitivity assays	- This technique requires that the supernatant must be removed from the drug assay without aspirating the schistosomula and the drug assay should be diluted to an acceptable fluorescence range as needed. These make the fluorometric L-lactate assay less than high-throughput
CellTiterGlo® (Commercial luminescence-based cell viability kit)	Detection of schistosomula viability through quantitation of ATP	- Suitable for a Medium-Throughput Assay semi-automated for drug screening - Fast, highly reliable, sensitive and automation friendly	- Require a precise multi-drop dispenser to ensure an exact number of NTS present in each well

**Table 4:** Summary of the published studies

Objective	Media used	NTS procedure	Outcome	Reference
Mansour et al. (2016) screened almost 300,000 compounds using an assay based on motility of worm, larvae and image analysis of assay plates	M169 supplemented with 100 U/ml Penicillin, 300 μg/ml Streptomycin, 0.25 μg/ml Fungizone (Amphotericin B) and 5 % fetal calf serum (FCS)	Mechanical transformation using the Syringe needle Method	A number of compounds were identified as promising leads for further chemical optimization	[54]
Panic et al. (2015a) investigate a panel of fluorescence/luminescence dyes for their applicability as viability markers in drug sensitivity assays for *Schistosoma mansoni* schistosomula	Medium 199 supplemented with 5 % heat iFCS and 1 % penicillin-streptomycin mixture	Mechanical in vitro transformation (vortexing)	Of the 11 markers selected for testing, resazurin, Vybrant® and CellTiter-Glo® correlated best with NTS viability, produced signals ≥ 3-fold stronger than background noise and revealed a significant signal-to-NTS concentration relationship	[23]
Panic et al. (2015b) expands the knowledge of antischistosomal properties of already approved 1600 FDA compounds from a very diverse set of indications against *Schistosoma mansoni*	Medium 199 supplemented with 5 % heat iFCS and 1 % penicillin/streptomycin	Mechanical in vitro transformation (vortexing)	Of the 1600 compounds screened against schistosomula, 121 were identified as active and 36 of these were active on the adult worms after Screening. The two in vivo- moderately active drugs identified in this study, doramectin and clofazimine present as novel drug classes as starting points for further investigation	[25]
Lalli et al. (2015) describes the development and validation of a luminescence based, medium-throughput assay for the detection of schistosomula viability through quantitation of ATP	DMEM complete tissue culture medium	Mechanical in vitro transformation (vortexing)	Schistosomula viability luminescence based assay is successful and suitable for the identification of novel compounds potentially exploitable in future schistosomiasis therapies Thus representing a valid alternative to fluorescence-based microscopy assays	[63]
Howe et al. (2015) assessed lactate as a surrogate marker for viability in *Schistosoma* drug screening assays by testing compounds with reported potencies	Phenol-red free medium 199 Supplemented with 5.5 mM D-glucose, 200 U/ml penicillin, 200 μg/ml streptomycin, 1 % heat iFCS	Mechanically transformed by vortexing	Lactate levels clearly reflected the viability of schistosomula and correlated with schistosomulum numbers. Lactate is a sensitive and simple surrogate marker to be measured to determine *Schistosoma* viability in compound screening assays	[24]
Ingram-Sieber et al.(2014) investigated the Medicines for Malaria Venture malaria box containing 200 diverse drug-like and 200 probe-like compounds with known antimalarial activity against larval stage of *S. mansoni*, followed by testing against adult worms in vitro and by in vivo studies of lead candidates	Supplemented Medium 199 with 5 % heat iFCS, penicillin (100 U/ml), and streptomycin (100 μg/ml)	Mechanically transformation by vortexing	Underlined the potential of compounds with an antimalarial background on schistosomes. Two entirely new chemical scaffolds with antischistosomal in vitro activity in the sub micromolar range and moderate in vivo activity identified	[12]
Protasio et al. (2013) analyzed differences in gene expression patterns between Mechanical and Skin Transformed *Schistosoma mansoni* Schistosomula and provide enough data to resolve a long-lasting controversy	Supplemented DMEM, 10 % FCS, 1 % Hepes buffer with 100 U/L penicillin, 0.1 mg/L streptomycin and 10 mM L-glutamine	- Mechanical transformation using the 21G Syringe needle method - Excised skin from mice	This work contributes to the validation of gene expression studies that have used Mechanical transformed schistosomula and provides further evidence that the MT is a good proxy for natural skin transformation	[37]
Coultas et al. (2012) compared a current and widely used double-ended-needle mechanical transformation method to a culture medium based on a nonmechanical method	RPMI 1640 medium enriched with L-glutamine; 150 units/ml penicillin, 100 μg/ml streptomycin and 5 % heat inactivated fetal bovine serum (iFBS)	Mechanical transformation using the 22-gauge double-ended, luer lok emulsifying needle	The mechanical and nonmechanical cercariae transformation methods both yielded significantly large and similar quantities of viable schistosomula	[49]
de Moraes et al. (2012) report the in vitro antischistosomal activity of piplartine on *S. mansoni* schistosomula of different ages	Basch 169 medium containing antibiotics and supplemented with 10 % fetal bovine serum (FBS)	Mechanical transformation, using a Vortex mixer	This report provides the first evidence that piplartine is able to kill schistosomula of different ages and reinforce that piplartine is a promising compound that could be used for the development of new schistosomicidal agent	[36]
Marxer et al. (2012) developed an in vitro drug screening assay for *S. haematobium* newly transformed schistosomula (NTS). The cercarial emergence rhythms of the intermediate hosts of *S. mansoni* and *S. haematobium, Biomphalaria glabrata* and *Bulinus truncatus* were studied, two artificial transformation methods for the production of the schistosomula were compared and the best purification method and optimal culture conditions were established by testing three different methods and several different media	Basch Medium 169, DMEM and Medium 199. All supplemented with 5 % heat iFCS and 200 U/ml penicillin and 200 μg/ml streptomycin	Mechanical transformation, using a Vortex mixer and chemical transformation using glucose	A circadian rhythm existed in both snail species. The highest transformation rate of *S. haematobium* cercariae into NTS was obtained with the vortex transformation and the highest purification factor was observed using Percoll. The fluorimetric readout based on resazurin was very precise in detecting dead schistosomula	[26]
Ingram et al. (2012) tested mefloquine-related compounds belonging to the three major groups of arylmethanols in order to elucidate their potential as antischistosomal lead candidates. The selected arylmethanols were tested against *S. mansoni* schistosomula and adults worms in vitro	Medium 199 supplemented with 5 % heat iFCS, penicillin (100 U/ml), and streptomycin (100 μg/ml)	Mechanically transformation by vortexing	The study confirmed the high antischistosomal activity of compounds with a mefloquine scaffold. Four candidates, WR7930, its two derivatives, and enpiroline, that are characterized by high antischistosomal properties in vivo were identified	[34]
Milligan et al. (2011) provide a visual description of cercarial transformation and in vitro culturing of schistosomules	RPMI 1640 supplemented with 5 % FBS, 1X Penicillin/Streptomycin	Mechanical transformation using the 22-gauge double-ended, luer lok emulsifying needle	This study developed a visual protocols for in vitro cercarial transformation and schistosomules culture techniques	[38]
Smout et al. (2010) describe a novel application for a real-time cell monitoring device (xCELLigence) that can simply and objectively assess anthelmintic effects by measuring parasite motility in real time in a fully automated high-throughput fashion	RPMI 1640, 1 % antibiotic/antimycotic and 10 mM Hepes	-	The study reported that the technique can be suitable for discovery and development of new anthelmintic drugs as well as for detection of phenotypic resistance to existing drugs for the majority of helminths and other pathogens where motility is a measure of pathogen viability	[62]
Mansour et al.(2010) report the development and validation of the Alamar Blue assay compared with morphology-based (microscopic) assessment of compound activity	M169 supplemented with 100 U/ml Penicillin, 100 mg/ml Streptomycin and 5 % FCS	Mechanical transformation using the Syringe Method	The Alamar Blue assay is readily able to detect compounds causing death or severe damage to the larvae but is less reliable than microscopy for more subtle morphological changes. It is concluded that an automated high throughput screen would benefit from integrated use of both alamar blue and automatic image-based morphology assays	[20]
Manneck et al.(2010) studied the temporal effect of this Mefloquine in vitro and in vivo, and examined alterations on the tegumental surface of schistosomula and adults of S. mansoni by means of scanning electron microscopy (SEM)	Basch medium 169 supplemented with 5 % heat iFCS and 100 U/ml penicillin and 100 mg/ml streptomycin	Mechanically transformation by vortexing	Mefloquine induces extensive morphological and tegumental alterations on both *S. mansoni* schistosomula and adults in vitro and in vivo	[27]
Peak et al. (2010) presented a microtiter plate-based method for reproducibly detecting schistosomula viability that takes advantage of the differential uptake of fluorophores (propidium iodide and fluorescein diacetate) by living organisms	DMEM lacking phenol red, containing 4500 mg/l glucose, supplemented with 10 % FCS, 2 mM L-glutamine, 200 U/ml penicillin, 200 mg/ml streptomycin	Mechanically transformation by vortexing	The study showed that developed method is sensitive (200 schistosomula/well can be assayed), relevant to industrial (384-well microtiter plate compatibility) and academic (96-well microtiter plate compatibility) settings, translatable to functional genomics screens and drug assays	[17]
Abdulla et al. (2009) presented a partially automated, three component phenotypic screen workflow that utilizes at its apex the schistosomula stage of the parasite adapted to a 96-well plate format. Hits that arise are subsequently screened in vitro against adult parasites and finally for efficacy in a murine model of disease	Basch Medium 169	Mechanical transformation using the 22-gauge double-ended, luer lok emulsifying needle	The study has identified various compounds and drugs as hits in vitro and leads, with the prescribed oral efficacy, in vivo	[18]

*NTS* newly transformed schistosomula, *DMEM* Dulbecco’s modified eagle’s medium, *RPMI* Roswell Park Memorial Institute, *iFCS* heat inactivated fetal calf serum, *SEM* scanning electron microscopy

## Conclusion

One of the great deal of new drug discovery against schistosomes is dependent on in vitro whole parasite screens. Access to adequate quantities and a substantial source of whole parasite organisms is a constant source of concern. Thus mechanically transformed schistosomula may be used as alternative for the purpose of high-throughput tools in the field of antischistosomal drugs discovery. This may help for the rapid discovery of urgently needed new drugs that will be useful in the control of schistosmiasis which represent one of the major devastating parasitic infections. Although it is clear from recent publications that subjective measures of NTS viability, motility and phenotype are still the gold standard, none of the automated assessment of phenotype have so far been validated.

## Abbreviations

AB: Alamar blue
DMEM: Dulbecco’s modified eagle’s medium
DMSO: Dimethyl sulfoxide
EB: Ethidium bromide
FBS: Fetal bovine serum
FDA: Fluorescein diacetate
HBSS: Hanks` balanced salt solution
IC: Inhibition concentration
iFCS: Inactivated fetal calf serum
MDA: Mass drug administration
MEM: Minimum essential medium
MMV: Medicines for malaria venture
NTS: Newly transformed schistosomula
PI: Propidium iodide
PZQ: Praziquantel
RPMI: Roswell Park Memorial Institute
TDR: Research and training in tropical diseases
